# Bone stress injury of the ankle in professional ballet dancers seen on MRI

**DOI:** 10.1186/1471-2474-9-39

**Published:** 2008-03-28

**Authors:** Ilan Elias, Adam C Zoga, Steven M Raikin, Judith R Peterson, Marcus P Besser, William B Morrison, Mark E Schweitzer

**Affiliations:** 1 Department of Orthopaedic Surgery, Rothman Institute, Thomas Jefferson University Hospital, 925 Chestnut Street, Philadelphia, PA 19107, USA; 2 Department of Radiology, Thomas Jefferson University Hospital, 111 S. 11th Street, Philadelphia, PA 19107, USA; 3 Department of Physical Therapy, Human Performance Laboratory, Thomas Jefferson University, 130 S. 9th Street, Philadelphia, PA 19107, USA; 4 Department of Radiology, Hospital for Joint Diseases, 301 East 17th Street, New York, NY 10003, USA

## Abstract

**Background:**

Ballet Dancers have been shown to have a relatively high incidence of stress fractures of the foot and ankle. It was our objective to examine MR imaging patterns of bone marrow edema (BME) in the ankles of high performance professional ballet dancers, to evaluate clinical relevance.

**Methods:**

MR Imaging was performed on 12 ankles of 11 active professional ballet dancers (6 female, 5 male; mean age 24 years, range 19 to 32). Individuals were imaged on a 0.2 T or 1.5 T MRI units. Images were evaluated by two musculoskeletal radiologists and one orthopaedic surgeon in consensus for location and pattern of bone marrow edema. In order to control for recognized sources of bone marrow edema, images were also reviewed for presence of osseous, ligamentous, tendinous and cartilage injuries. Statistical analysis was performed to assess the strength of the correlation between bone marrow edema and ankle pain.

**Results:**

Bone marrow edema was seen only in the talus, and was a common finding, observed in nine of the twelve ankles imaged (75%) and was associated with pain in all cases. On fluid-sensitive sequences, bone marrow edema was ill-defined and centered in the talar neck or body, although in three cases it extended to the talar dome. No apparent gender predilection was noted. No occult stress fracture could be diagnosed. A moderately strong correlation (phi = 0.77, p= 0.0054) was found between edema and pain in the study population.

**Conclusion:**

Bone marrow edema seems to be a specific MRI finding in the talus of professional ballet dancers, likely related to biomechanical stress reactions, due to their frequently performed unique maneuvers. Clinically, this condition may indicate a sign of a bone stress injury of the ankle.

## Background

Bone marrow edema (BME) is a common finding on magnetic resonance images (MRI) of the ankle; this finding has a wide diagnostic differential, including etiologies such as trauma [1-5], avascular necrosis [6,7], osteochondral defect [8,9], tumors and tumor-like conditions [10,11], metabolic disease [12], tarsal coalition [13,14], infection [15-18], arthritis [17-21], as well as tendinopathy [22] and plantar fasciitis [23]. Although bone marrow edema is generally associated with pathology, asymptomatic edema-type patterns related to long-distance running and altered biomechanics have been identified on MR images of the ankle in prior reports [24-26]. We sought to investigate whether this effect would also be observed in a population whose occupation involves specific, repetitive lower extremity biomechanical stresses and who frequently perform full weight bearing maneuvers in extensive plantar flexion, such as on pointes and demi-pointes maneuvers.

In this study, it was our objective to look at MRI patterns of bone marrow edema in the ankles of professional high performance ballet dancers and to evaluate their clinical relevance.

## Methods

Twelve ankles of 11 professional ballet dancers (6 female, 5 male, mean age 24 years, range 19 to 32) were MR imaged after obtaining approval from the institutional review board (IRB) at our institution. All subjects have agreed to participate in the study and have signed the informed written consent.

Individuals participating in the study were recruited by the team physician caring for the local professional ballet company. All available ballet dancers were recruited regardless of symptoms. They were all actively ballet dancing without limitation; nevertheless, all were questioned as to whether they experienced any ankle pain at rest or during activity.

Individuals were imaged on a 0.2 T extremity MRI unit (10 ankles; Artoscan, Esaote) or 1.5 T MRI units (2 ankles; GE Medical Systems). For all ankles, images were obtained in sagittal, coronal and axial planes. For the 0.2 T unit, the protocol consisted of sagittal and axial T1-weighted spin echo (TR/TE = 380/18), axial T2-weighted spin echo (TR/TE = 2360/80) and sagittal and coronal short inversion time inversion recovery (STIR, TR/TE/TI = 1260–1800/24/75) imaging sequences using 2–3 excitations. Slice thickness was 4–5 mm with an interslice gap of 1 mm. Field of view was 16–20 cm; matrix size was 256 by 128 or 192.

For studies performed in the 1.5 T unit, an extremity coil was used. The protocol consisted of sagittal T1-weighted spin echo (TR/TE = 400–700/10–18), axial and coronal fat-suppressed T2-weighted fast spin echo (TR/TE = 2000–7000/60–80 effective, echo train length = 8) and sagittal STIR (TR/TE/TI = 3500–5000/36–75/150) imaging sequences using 2–3 excitations. Slice thickness was 4–5 mm with an interslice gap of 1 mm. Field of view ranged from 14 cm to 18 cm; matrix was 256 by 192. Fat suppression for fast spin echo T2-weighted images was achieved with selective presaturation of lipid resonant frequency.

Images were reviewed by two musculoskeletal radiologists and one orthopaedic surgeon in consensus. The three reviewers of the MRI studies were blinded to the pain questionnaire. Bone marrow signal was evaluated for presence of edema-type signal (high signal on STIR or fat-suppressed T2-weighted images). If edema-type marrow signal was present, the location and morphology (poorly defined or sharply defined, rounded or linear) were recorded, and the T1 signal (relative to fat and muscle) in the area was noted.

In order to control for recognized sources of bone marrow edema, images were also reviewed for presence of a talar dome osteochondral defect (defined as focal subchondral marrow edema) or osteoarthritis of the tibiotalar, subtalar, Chopart or intertarsal joints (defined as osteophytes at the joint margin or focal areas of subchondral edema on both sides of a joint); if such subchondral marrow edema was present, the location was recorded. Presences of accessory ossicles were recorded along with any adjacent bone marrow edema. Bone marrow edema has also been reported in association with ligament disruption [1-3], medial and lateral tendinopathy (in a subtendinous location) [22], and plantar fasciitis (in the calcaneus at the plantar fascia origin) [23]. Therefore, edema was also recorded if focally present in association with anterior talofibular ligament tear, in a subtendinous location or at the plantar fascia origin. Tendons were evaluated for internal signal or surrounding sheath fluid on T2-weighted or STIR images. The plantar fascia was evaluated for internal signal or surrounding soft tissue edema on sagittal T2-weighted or STIR images. The anterior talofibular ligament was evaluated as intact or disrupted on axial images. Statistical analysis was performed to assess the strength of the correlation between bone marrow edema and ankle pain.

## Results

Although all dancers were actively training and performing without limitation, nine of eleven claimed to experience mild ankle pain during activity; five of these individuals also reported some pain at rest.

All tendons were normal based on morphology and signal characteristics. One ankle had a moderate amount of fluid in the flexor hallucis longus tendon sheath. One ankle had a small accessory navicular ossicle, but without adjacent bone marrow edema.

There was evidence of prior anterior talofibular ligament tear in two individuals (seen as thickening of the ligament without discontinuity); one had intermediate signal at the anterolateral gutter suggesting anterolateral impingement; there was no adjacent bone marrow edema in these individuals. One individual had a moderate inferior calcaneal enthesophyte, but no edema at the plantar fascia to suggest plantar fasciitis, and no adjacent bone marrow edema.

Two individuals had anterior ankle joint spurring suggesting anterior osseous impingement. However, these asymptomatic individuals did not have evidence of cartilage loss or subchondral cystic change to suggest osteoarthritis. No other articular abnormalities were seen. There was no evidence of osteochondral defect or stress fracture in any of the twelve ankles. There were no significant joint effusions.

One individual had an os trigonum without evidence of surrounding edema or fluid to suggest os trigonum syndrome.

**Figure 1:**
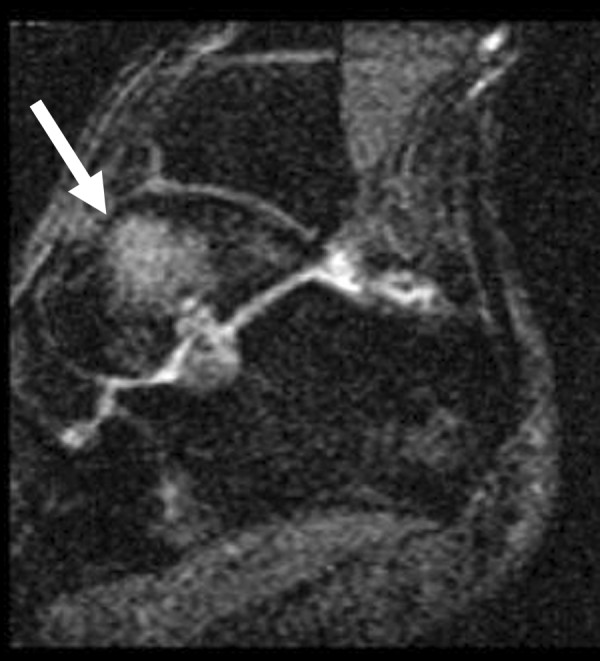
Typical appearance of talar bone marrow edema observed on MR images of nine out of twelve ankles of symptomatic professional ballet dancers: Sagittal STIR (TR/TE = 1800/24) image of a 24-year-old male ballet dancer shows associated edema-type high signal within the talus (arrow).

**Figure 2:**
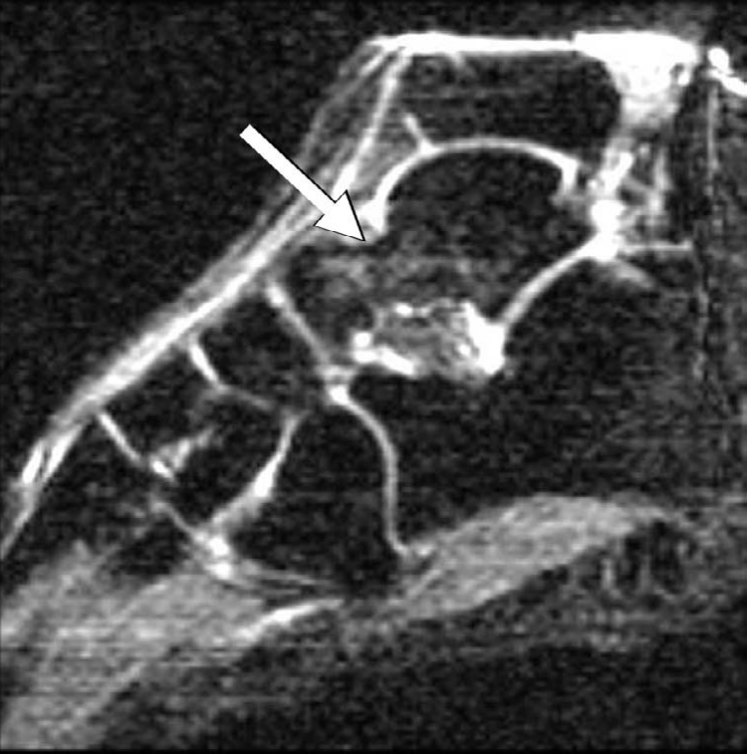
Sagittal STIR MR image of a 32-year-old female shows patchy edema signal within the talar neck (arrow).

**Figure 3:**
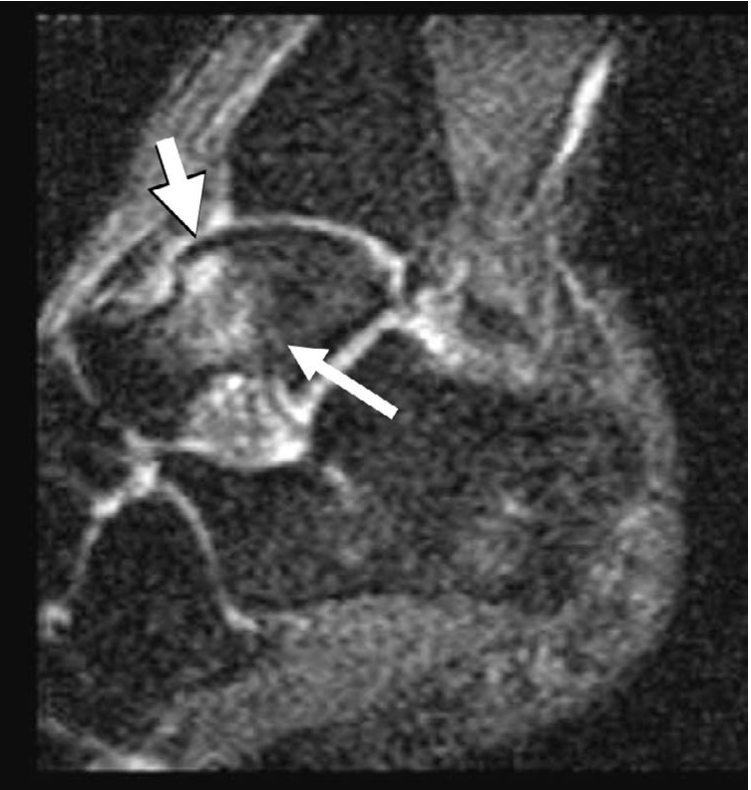
Sagittal STIR MR image of a 25-year-old male shows patchy edema signal within the body of the talus (long arrow), which extends to the subchondral region of the talar dome (short arrow). This same edema pattern was observed in nine of the twelve ankles and may be related to chronic repetitive stress.

**Figure 4:**
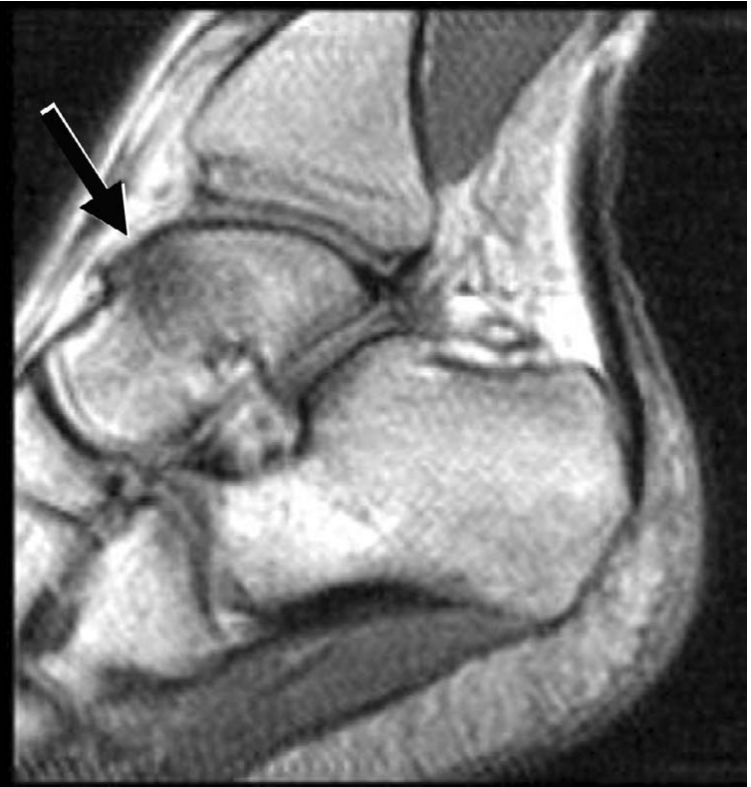
Talar bone marrow edema in a symptomatic 24-year-old male (same subject as in figure 1). Sagittal T1-weighted (TR/TE = 380/18) MR image shows patchy low signal within the talus (arrow).

Bone marrow edema signal was seen on fluid-sensitive sequences in nine of the twelve ankles (75%); in all cases, the edema was within the talus, centered in the talar neck or body (Figure 1). In three cases the edema extended toward the talar dome but was clearly centered in the neck (Figure 2) or body (Figure 3) of the talus. Morphologically, the edema was patchy with a rounded shape and ill-defined margins in all cases. The regions of marrow edema also showed patchy low signal (lower than surrounding fatty marrow, but higher than adjacent muscle) on T1-weighted images (Figure 4). All subjects presented with marrow edema strictly located in the talus, which was not seen in other bones of the ankle or foot.

There were five females with this edema pattern, and three males (one male had bilateral talar edema). All eight patients (nine ankles) with marrow edema had associated pain. Of the three patients without evidence of marrow edema, one female was with pain and two males were without pain.

A phi correlation was run to assess the association of bone marrow edema and ankle pain [27]. Eleven ankles were analyzed (the male subject with bilateral pain was treated as a single independent datapoint for this analysis). See Table 1.

**Table 1:** Correlation of bone marrow edema and ankle pain.

	**Pain**	**No pain**
Bone Marrow Edema	8 (A)	0 (B)
No Bone Marrow Edema	1 (C)	2 (D)

(n = 11)

Phi was calculated as follows:

$$\begin{array}{l} \varphi =\frac{BC-AD}{\sqrt{\left(A+B)(C+D)(A+C)(B+D\right)}}=\frac{\left(0)(1)-(8)(2\right)}{\sqrt{\left(8+0)(1+2)(8+1)(0+2\right)}}=0.7698\\ z=\varphi \sqrt{n}=0.7298\;\sqrt{11}=2.5531;\;\;\text{area above z}=\text{0}\text{.0054} \end{array}$$

A moderately strong correlation (phi = 0.77, p= 0.0054) was found between edema and pain.

## Discussion & Conclusion

Professional ballet dancers undergo intensive training, with specific maneuvers that expose their ankles to tremendous and somewhat unique stresses. Additionally, different maneuvers and positions performed by male and female dancers result in different, gender-specific stresses that could potentially be used to study the body's response to specific stress.

In this albeit small population of professional ballet dancers, we saw very little evidence of chronic injury patterns (tendinopathy, ligament tear or scarring, arthritis) which would be expected in a population of active, high-performance athletes. This may be due to a selection bias; the precision required to perform advanced ballet maneuvers may exclude individuals with a predisposition to ankle pathology before they attain professional status. Secondary gain is a possible source of bias that could affect the results of this study; as with other professional athletes, individuals in our population might potentially hide symptoms in order to avoid being placed on inactive status.

A large proportion (n = 9, 75%) of ankles in our population showed a patchy pattern of marrow edema within the talus. The edema was centered in the talar neck and body, although in some cases it extended to the talar dome or head. The uniqueness of this finding was that no case showed corresponding subarticular marrow edema signal in the tibia, calcaneus or navicular bone, and there were no discrete subchondral cysts. This argues against osteoarthritis or other articular process as an etiology. The edema was not centered in the subchondral bone, but rather predominated in the talar neck and body. This distribution and lack of pain at rest argues against osteochondral defect as well as avascular necrosis as an etiology. In addition, there was no 'double line sign' detected in the area of edema. The edema pattern was non-linear, without discrete form, which in conjunction with lack of symptoms also argues against stress fracture. This non-linear pattern also argues against prominent vascular channels simulating bone marrow edema.

We assume that the etiology for the finding relates to subclinical injury, trabecular reorganization in response to stress, or probably vascular proliferation in an area chronically undergoing excess stress.

However, we explain that the most likely extensive axial load-type stress on the talus, which occurs in full-weight bearing plantar flexion (for example in en-pointes and demi-pointes positions), can potentially cause overload stresses on the anatomical structures (trabecular system, vascular system) of the bone. This may result in a pathophysiologic transudation process of the intraosseous vessels, or produces trabecular re-organization, which is then seen as bone marrow edema on MR imaging.

A spectrum of osseous stress injury of the talus has been reported by Sormaala et al. involving both the talar dome and the talar head, with stress fracture and subchondral depression representing the most severe pathology. [28,29]

In our patient population, a group exposed to extreme biomechanical stresses and predisposed to osseous stress injury, identification of osseous injury early in the disease process may help to prevent evolution to fracture and ultimately articular collapse or arthropathy.

We are not aware that any changes to the training regimen were required in dancers who had the edema pattern versus those that did not. Since all subjects with BME had associated ankle pain and a moderate strong phi correlation was found between BME and pain in our entire study population, this finding is likely to be useful as a clinical marker for over-training and may indicate an 'overuse syndrome'. However, this finding should not be misinterpreted as pathology, such as occult fracture, unless it is recognized as an incidental finding.

Reviewing the literature, we could not find any study reporting BME of the ankle in ballet dancer associated with pain. For example, Bronner, et al. [30] and Kadel, et al. [31] described that ballet dancers have a relatively high incidence of stress fractures of the lower extremities. However, these articles did not point out the occurrence of bone marrow edema associated with stress fractures.

Menetrey, et al [32] reported that subtalar subluxation have been diagnosed in 10.5% of all injuries of 60 ballet dancers within one season. Associated with this injury the talonavicular joint was tender and swollen in the acute phase. This is a hint that the talonavicular joint in ballet dancers can be over utilized and therefore susceptible for stress reactions. Nevertheless, the authors did not discuss any bone marrow edema pattern of the ankle on MRI.

Schweitzer, et al. [26] reported foot and ankle marrow edema patterns in asymptomatic people undergoing altered biomechanical stress. Lazzarini, et al. [24] and Lohman, et al. [25] have reported bone marrow edema patterns in the foot and ankle of active people without symptoms of stress fracture, especially in runners. These three articles, which represent the only reports of this phenomenon, describe different locations and morphology of bone marrow edema in their populations; none describe the consistent pattern of talar bone marrow edema-type signal that was common in our population.

This talar edema pattern could be due to the specific ankle stresses unique to ballet dancing.

Peace et al. reported of MRI features of posterior ankle impingement syndrome in 25 ballet dancers. The authors found bone marrow edema in all patients. The pattern occurred commonly in the posterior corner of the talus and was in most cases also distributed to the adjacent talar bones [33].

There were some limitations to this study. None of the individuals underwent follow-up imaging to observe changes in this pattern over time.

This study did not have a control group for example an age matched group of dancers with no pain because there were not available to us. The use of 0.2 T MRI may have limitations in the imaging quality. However, 0.2 T MRI is a valuable tool in identifying and assessing bone marrow alterations [34].

In summary, tendon and ligament abnormalities were unusual in our population of professional ballet dancers. However, most demonstrated a pattern of edema-type signal in the talus, interpreted as bone marrow edema, which is related to occupational repetitive biomechanical stress reactions in the high-performing ballet dancer.

The bone marrow edema pattern we described in this study should not be misinterpreted as a morphologic pathology like stress fracture, avascular necrosis, osteochondrosis dissecans or degenerative arthritis.

We consider this specific finding to be a sign for an overuse syndrome and may indicate an earlier and less severe bone stress injury of the talus. Therefore, the appearance of bone marrow edema within the body of the talus on MRI, when symptomatic, should suggest to the sports physician to reconsider the training regimen and probably advise the ballet dancer to reduce training intensity.

## Competing interests

The author(s) declare that they have no competing interests.

## Authors' contributions

IE and ACZ have prepared the manuscript. SMR and WBM have reviewed the manuscript. JRP recruited the subjects. MPB did the statistics. MES has reviewed the manuscript and did the study design.

## Pre-publication history

The pre-publication history for this paper can be accessed here:


